# Roles Played by the PI3K/Akt/HIF-1*α* Pathway and IL-17A in the Chinese Subtype of Chronic Sinusitis with Nasal Polyps

**DOI:** 10.1155/2022/8609590

**Published:** 2022-01-15

**Authors:** Ke-Jia Cheng, Min-Li Zhou, Yong-Cai Liu, Shui-Hong Zhou

**Affiliations:** Department of Otolaryngology, The First Affiliated Hospital, School of Medicine, Zhejiang University, Hangzhou, China

## Abstract

**Background:**

The Chinese subtype of CRSwNP may have a unique pathogenesis. This study was designed to seek the role of the PI3K/Akt/HIF-1*α* pathway and IL-17A in CRSwNP.

**Methods:**

The total IgE, ECP, and IL-17A levels were determined by UniCAP100 and ELISA. The activity of MPO was detected by the biochemical techniques. The protein expressions of HIF-1*α*, p-Akt, and PI3K were detected by the WB method. HIF-1*α* and IL-17A mRNA levels were measured by RT-PCR.

**Results:**

The CRSwNP group showed significantly elevated MPO activity, PI3K, p-AKT protein, HIF-1*α*, and IL-17A mRNA levels in nasal polyps. Stimulated by the TNF-*α*, the PI3K, p-AKT, HIF-1*α*, and IL-17A levels significantly elevated in the fibroblasts. Inhibited by the Wortmannin, those indicators significantly declined in the fibroblasts.

**Conclusion:**

The PI3K/Akt/HIF-1*α* pathway played a role in the pathogenesis of CRSwNP. The elevated IL-17A level might be responsible for the neutrophilic inflammation in CRSwNP. The PI3K/Akt/HIF-1*α* pathway might regulate the IL-17A-related inflammation in CRSwNP.

## 1. Introduction

Chronic sinusitis (CRS) is divided into CRS with nasal polyps (CRSwNP) and CRS without nasal polyps (CRSsNP) [1]. The precise etiology of CRSwNP has not been fully elucidated. It is currently believed that allergies, infections, inflammation, anatomical abnormalities, genetic factors, microbial superantigens, and bacterial biofilms affect the pathogenesis. Depending on the infiltrating inflammatory cells, CRSwNP can be divided into eosinophilic and noneosinophilic subtypes. The etiologies, clinical manifestations, and required treatments differ. Here, we preliminarily differentiated the subtypes of CRSwNP by measuring the levels of eosinophil cationic protein (ECP) and myeloperoxidase (MPO) in nasal polyps.

Hypoxia plays an important role in the pathogenesis of CRS and nasal polyp formation [2]. During hypoxia, the body activates many regulatory factors to maintain an appropriate intercellular oxygen concentration. Of these, hypoxia-inducible factor-1*α* (HIF-1*α*) is closely related to the development of CRSwNP. HIF-1*α* is expressed by macrophages, neutrophils, dendritic cells, and lymphocytes and is a core component of the hypoxia response. HIF-1*α* plays a role in the pathogenesis of nasal polyps by regulating the expression of downstream factors, increasing vascular permeability and tissue edema, and promoting the transformation of epithelial cells into mesenchymal cells. HIF-1*α* is regulated by the phosphoinositide 3-kinase (PI3K)/protein kinase B (Akt)/mammalian target of rapamycin (mTOR) pathway that controls several cellular processes including metabolism, inflammation, survival, division, and cancer progression [3]. However, any role for the PI3K/Akt/HIF-1*α* pathway in CRSwNP development remains unclear. Here, we measured both the protein levels and the levels of mRNA encoding of HIF-1*α*, PI3K, and Akt in nasal polyps.

Th17 cells are a subgroup of CD4^+^ T cells that were recently described and are characterized by the production of IL-17A, which plays an important role in airway inflammation and perhaps in noneosinophilic CRSwNP development [4]. However, the latter role of IL-17A remains controversial. We explored the role of IL-17A in the pathogenesis of CRSwNP.

## 2. Materials and Methods

This study was approved by the Scientific Research Ethics Review Committee of the First Affiliated Hospital of Zhejiang University Medical College.

### 2.1. Patients

We retrospectively analyzed the data on 53 patients with CRSwNP who underwent endoscopic sinus surgery in our hospital from January to March 2017. CRSwNP was diagnosed using the EPOS 2012 guidelines for the diagnosis and treatment of chronic rhinosinusitis [5]. No nasal decongestants or antihistamines were administered for 4 weeks prior to surgery. The nasal tissues of 17 patients without allergic disease who underwent surgery to remove sinus cysts served as the control tissues. The preoperative computed tomography (CT) sinus score was obtained using the Lund-MacKay criteria.

### 2.2. Nasal Supernatants

Nasal tissues obtained during surgery were immediately frozen in liquid nitrogen and stored at -80°C. First, the nasal tissues were thawed and chopped. Then normal saline was added (1 g/10 mL), and the tissues were homogenized. The suspensions were centrifuged at 3,000 RPM at 4°C for 5 min, and the supernatants were stored at 4°C.

### 2.3. Allergy Test

The Phadiatop system was used to detect systemic allergies, and the UniCAP100 system was used to measure the serum levels of the total IgE and ECP following the directions of the manufacturer (Famasia, Sweden) and the literature.

### 2.4. Enzyme-Linked Immunosorbent Assays (ELISAs)

ELISAs were used to measure the total IgE and ECP levels in nasal homogenate supernatants (Cloud-Clone, USA). Briefly, the experimental procedure was as follows. Samples were added to the plate and coating proceeded at 37°C for 2 h. The liquid was discarded and the plate was dried. Test solution A (100 *μ*L) was added to each well, the labeled plate was covered, and incubation at 37°C for 1 h followed. The liquid was discarded, and the plate was washed three times. Test solution B (100 *μ*L) was added to each well, and incubation at 37°C for 30 min followed. The liquid was discarded and the plate was washed five times. Substrate solution (90 *μ*L) was added to each well, and incubation at 37°C for 25 min followed. Termination solution (50 *μ*L) was added to each well, the optical density at 450 nm was measured using a microplate reader, and sample concentrations were calculated.

### 2.5. Biochemical Method

A biochemical method was used to detect the myeloperoxidase (MPO) activity in the supernatants of nasal tissue homogenates, following the manufacturer's instructions (China Jiancheng Co., China).

### 2.6. Western Blotting (WB)

WB was used to detect HIF-1*α*, p-Akt, and PI3K in nasal tissues. The anti-GAPDH and anti-HIF-1*α* antibodies were provided by Bioworld (USA). Anti-p-Akt and anti-PI3K antibodies were from CST (USA). Briefly, the tissue was ground and dissolved, incubated on ice for 30 min, and centrifuged at 12,000 rpm for 15 min at 4°C. The BCA Protein Assay Kit was used to detect protein concentration. Proteins were tested by sodium dodecyl sulfate-polyacrylamide gel electrophoresis and electrotransferred to membranes. The membranes were incubated overnight at 4°C with primary antibody. Then, the membranes were washed five times with TBST and then incubated with a secondary antibody at 37°C for 2 h. After that, the membrane was washed again five times with TBST. Proteins were visualized by enhanced chemiluminescence.

### 2.7. Real-Time Quantitative Reverse Transcription Polymerase Chain Reaction (RT-PCR)

The levels of nasal tissue mRNA encoding HIF-1*α* and IL-17A were detected via RT-PCR. The reverse transcription kit and the RevertAid first-strand cDNA Synthesis Kit were provided by Thermo Scientific Fisher (USA). The qPCR kit SuperReal PreMix Plus was that of Tiangen (China). The total RNA rapid extraction reagent was purchased from Shanghai Jierui Biotechnology Co. (China). The experimental procedures were those of the manufacturers' instructions. Briefly, the total RNA was isolated from nasal tissues. To generate first-strand cDNA, samples were treated with DNase I to remove DNA and subjected to reverse transcription in reactions containing RNA-Primer Mix, 25 mM dNTPs, 25 U/*μ*L RNase inhibitor, 200 U/*μ*L M-MLV reverse transcriptase, oligo, and DNase-free water. The HIF-1*α* primers were sense 5′-TTTTGGCAGCAACGACACAG-3′ and antisense 5′-GTGCAGGGTCAGCACTACTT-3′. The IL-17A primers were sense 5′-CGATCCACCTCACCTTGGAA-3′ and antisense 5′-GTGCAGGGTCAGCACTACTT-3′. The primers for Actin (control) were sense 5′-AGCGAGCATCCCCCAAAGTT-3′ and antisense 5′-GGGCACGAAGGCTCATCATT-3′. Real-time PCR was performed at 50.0°C for 3 min, 95.0°C for 15 min, 95.0°C for 10 s, 59.0°C for 20 s, and b72.0°C for 30 s, plate read, GOTO 2 (45 times), melt cure 65°C to 95°C, increment 0.5°C for 10 s, and plate read.

### 2.8. Experiments In Vitro Using Fibroblasts from Nasal Polyps

#### 2.8.1. Isolation and Culture of Fibroblasts

Fibroblasts were isolated from the polyps of five patients with CRSwNP. Removed polyps were immediately placed into phosphate buffer saline (PBS) at 4°C and repeatedly washed with PBS in Petri dishes placed on an ultraclean table to remove red blood cells and secretions. The polyps were cut into 1–2 mm cubes and digested with collagenase, hyaluronidase, and DNase. The suspensions were sieved through a 250-mesh sieve, transferred to bottles, and cultured in an incubator. When cells covered the bottoms of the bottles, digestion was performed. Under the microscope, fibroblasts became rounded and partially detached. The cell suspensions yielded by digestion were collected and centrifuged, resuspended in serum-free DMEM medium, and inoculated into fresh culture flasks. After 20 min, most cells were attached to the walls, and we carefully removed the medium. DMEM with penicillin/streptomycin and 10% (*v*/*v*) FBS was added to allow further culture.

#### 2.8.2. Fibroblast Identification and Proliferation

Fibroblast morphology was observed under a light microscope. Vimentin, Thy-1, and E-cadherin were detected via immunohistochemistry (IHC). Proliferation was measured using the 3-(4,5-dimethylthiazole-2)-2,5-diphenyl tetrazole-bromide (3-(4,5)-dimethylthiahiazoazo(-z-y1)-3,5-di-phenytetrazoliumromide) (MTT) method (Sigma, USA).

#### 2.8.3. Experimental Groups

The experimental groups were divided into controls (untreated fibroblasts), the nasal polyp fibroblasts + TNF-*α* group (fibroblasts treated with 10 ng/mL TNF-*α* for 10 h), and the nasal polyp fibroblasts + TNF-*α* + Wortmannin group (fibroblasts treated with 10 ng/mL TNF-*α* for 10 h and 2 *μ*mol/L Wortmannin for 12 h).

#### 2.8.4. The Levels of PI3K/Akt/HIF-1*α* and IL-17A in the Fibroblasts

Fibroblast levels of HIF-1*α*, p-Akt, and PI3K were detected by WB (Abcam, UK). Levels of IL-17A were measured via ELISA (Mersak Biotechnology Co. Ltd., China). The levels of mRNA encoding HIF-1*α* and IL-17A were detected via RT-PCR (Beskey Technology Co. Ltd., China). The procedures were the same as described above.

### 2.9. Statistical Analyses

The SPSS ver. 20.0 software was used for all statistical analyses. Fisher's exact test was employed to compare between-group data. The chi-square test was used to analyze the differences in gender and age between the patients and controls. By the test of SPSS, the data had an abnormal distribution. We used medians (with 25–75% ranges) to present the data. The Mann–Whitney *U*-test was employed to compare between-group differences. The Kendall test was used to seek correlations between indices. The paired *t*-test was employed to analyze the differences between the fibroblast groups. A *P* value < 0.05 was considered statistically significant.

## 3. Results

The 53 patients with CRSwNP included 32 males and 21 females of average age 45.2 years and a disease duration of 3 months to 40 years. Five patients had asthma, and nine had histories of nasal surgery. There was no significant difference in gender and age between the patients with CRSwNP and controls.

### 3.1. Inflammatory Indicators in Serum and Nasal Polyps

**Figure 1:**
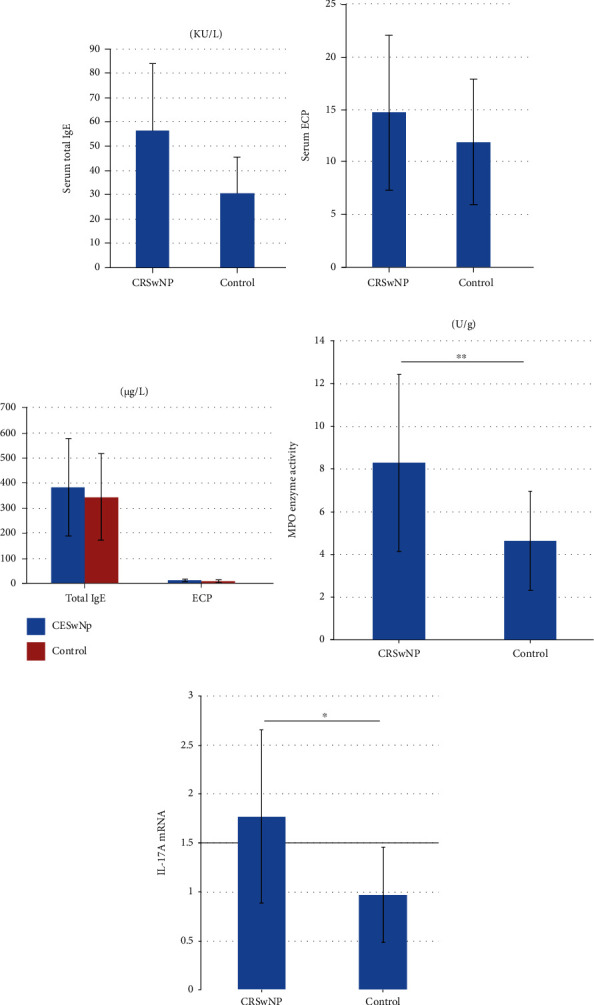
The levels of inflammatory indicators in serum and nasal polyps in different groups. (a) By the UniCAP100 system, the serum levels of total IgE did not significantly differ between CRSwNP patients and controls. *P* = 0.451. (b) By the UniCAP100 system, the serum ECP levels did not significantly differ between CRSwNP patients and controls. *P* = 0.058. (c) By ELISAs, the total IgE and ECP levels in nasal polyp supernatants of CRSwNP patients were not significantly different from those observed in control. *P* = 0.547 and 0.132. (d) By the biochemical method, the enzyme activity of MPO in nasal polyps of CRSwNP patients was higher than that in control tissues. *P* < 0.001. (e) By RT-PCR, the level of IL-17A mRNA in nasal polyps of CRSwNP patients was significantly increased, compared with control tissues. *P* = 0.049. ^∗^*P* < 0.05, ^∗∗^*P* < 0.001.

The Phadiatop allergen screening test (via inhalation) was positive in six patients; all controls were negative (*P* < 0.001). The serum levels of total IgE and ECP did not significantly differ between CRSwNP patients and controls (Figures 1(a) and 1(b)), nor did they in nasal polyp supernatants (Figure 1(c)). The nasal polyp groups had higher MPO activities than controls (Figure 1(d)). The level of mRNA encoding IL-17A was significantly higher in the nasal polyp groups than in controls (Figure 1(e)).

### 3.2. PI3K/Akt/HIF-1*α* Pathway Activity in Nasal Polyps

**Figure 2:**
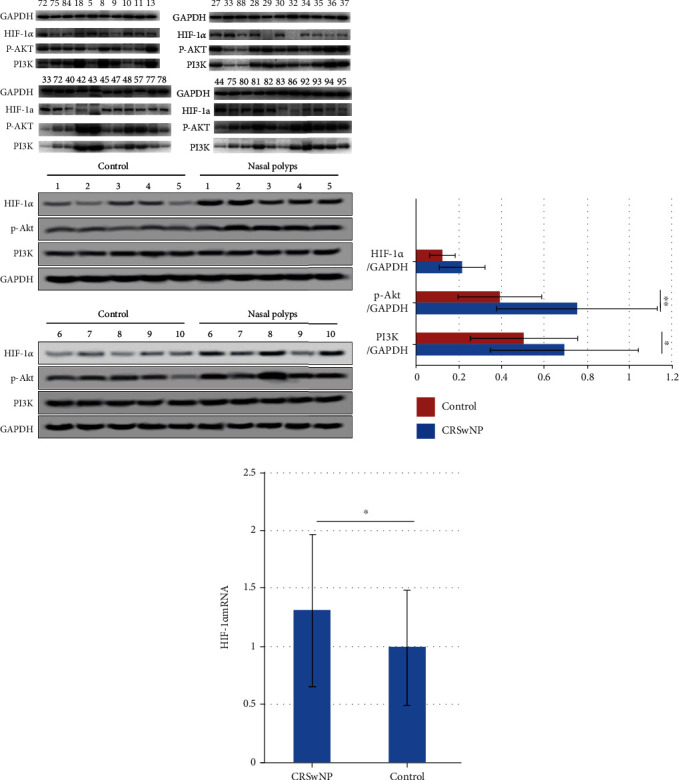
The activity of the PI3K/Akt/HIF-1*α* pathway in nasal polyps. (a) The protein levels of HIF-1*α*, p-Akt, and PI3K were detected by the WB method. Among them, 27, 33, 44, 72, 75, 81, 84, 88, and control 1-10 were the control groups, and the rest were the experimental groups. (b) The protein levels of HIF-1*α*, p-Akt, and PI3K in different groups. Among them, p-Akt and PI3K protein levels in the polyp group were significantly increased, compared with the control group. *P* < 0.001 and *P* = 0.008. The HIF-1*α* protein level was not significantly different from that observed in control tissues. *P* = 0.242. (c) The HIF-1*α* mRNA expression level in nasal polyp tissues of CRSwNP patients was significantly higher than that in the control group. *P* = 0.012. ^∗^*P* < 0.05, ^∗∗^*P* < 0.001.

The protein levels of HIF-1*α*, p-Akt, and PI3K in nasal polyps were measured via WB (Figure 2(a)). The HIF-1*α* protein level did not differ from that of controls, but the levels of p-Akt and PI3K were significantly higher than those of controls, as was the level of mRNA encoding HIF-1*α* (Figures 2(b) and 2(c)).

### 3.3. Correlations among CT Scores, Serum, and Tissue Indicators in CRSwNP Patients

We found a significant positive correlation between total IgE levels in the serum and supernatant (*r* = 0.301, *P* = 0.001). The p-Akt and PI3K levels were significantly (positively) correlated in nasal polyps (*r* = 0.755, *P* < 0.001), as were those of HIF-1*α* and p-Akt (*r* = 0.408, *P* < 0.001) and HIF-1*α* and PI3K (*r* = 0.386, *P* < 0.001), and the level of mRNA encoding HIF-1*α* and those encoding p-Akt (*r* = 0.397, *P* < 0.001), PI3K (*r* = 0.438, *P* < 0.001), and HIF-1*α* (*r* = 0.415, *P* < 0.001). We found no other significant correlations (Table 1).

Sinus CT scores were significantly correlated with serum total IgE in CRSwNP patients (*r* = 0.199, *P* = 0.041). There was no significant correlation between sinus CT scores and other indicators.

### 3.4. In Vitro Fibroblast Experiments

#### 3.4.1. Identification and Proliferation of Fibroblasts from Nasal Polyps

**Figure 3:**
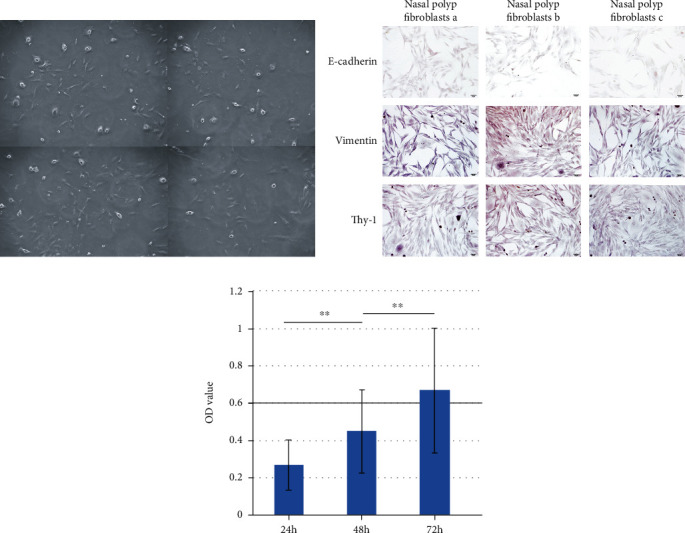
Identification and proliferation of fibroblasts from nasal polyps. (a) Morphology of fibroblasts under light microscope. (b) Expression of vimentin, Thy-1 protein, and E-cadherin protein in fibroblasts. (c) Cell activity increased and cell proliferation gradually increased with time. ^∗∗^*P* < 0.001.

Fibroblast morphology was observed under a light microscope (Figure 3(a)). Vimentin, Thy-1, and E-cadherin were detected via IHC (Figure 3(b)). The MTT assay showed that the optical density was significantly higher after 48 h of culture than after 24 h of culture and increased further (with significance) to 72 h of culture (Figure 3(c)). Thus, fibroblast culture was successful; cell activity and proliferation increased gradually over time.

#### 3.4.2. Expression of the PI3K/Akt/HIF-1*α* Pathway and IL-17A in Fibroblasts

**Figure 4:**
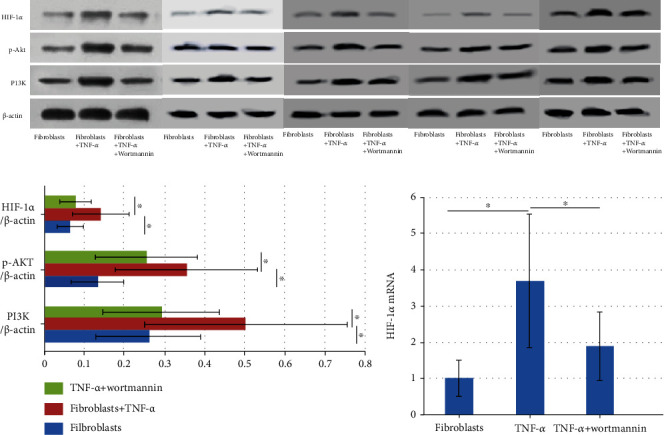
The expressions of the PI3K/Akt/HIF-1*α* pathway in fibroblasts from nasal polyps. (a) The protein levels of HIF-1*α*, p-Akt, and PI3K in nasal polyp fibroblasts were detected by the WB method. (b) After TNF-*α* stimulation, the HIF-1*α*, p-Akt, and PI3K protein levels were significantly increased in nasal polyp fibroblasts. *P* = 0.032, 0.032, and 0.032. After treatment with Wortmannin, the HIF-1*α*, p-Akt, and PI3K protein levels were significantly decreased in nasal polyp fibroblasts. *P* = 0.032, 0.008, and 0.032. (c) Expression of HIF-1*α* mRNA in fibroblasts of nasal polyps. After TNF-*α* stimulation, the HIF-1*α* mRNA level was significantly increased in nasal polyp fibroblasts. *P* = 0.001. After treatment with Wortmannin, the HIF-1*α* mRNA level was significantly reduced in nasal polyp fibroblasts. *P* = 0.001. ^∗^*P* < 0.05.

After TNF-*α* stimulation, the HIF-1*α* concentration in fibroblasts, the levels of p-Akt, and PI3K in nasal polyp fibroblasts, and the level of mRNA encoding HIF-1*α* all significantly increased. In all cases, the levels significantly decreased after treatment with the PI3K inhibitor Wortmannin (Figures 4(a)–4(c), Table 2).

**Figure 5:**
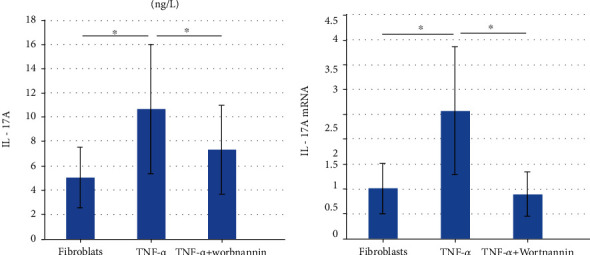
The expressions of IL-17A in fibroblasts from nasal polyps. (a) By ELISAs, after TNF-*α* stimulation, the IL-17A level in nasal polyp fibroblasts was significantly increased. *P* = 0.006. After treatment with Wortmannin, the IL-17A level in nasal polyp fibroblasts was significantly reduced. *P* = 0.014. (b) By RT-PCR, after TNF-*α* stimulation, expression of IL-17A mRNA in fibroblasts of nasal polyps was significantly increased. *P* = 0.049. After treatment with Wortmannin, the IL-17A mRNA level in nasal polyp fibroblasts was significantly reduced. *P* = 0.015. ^∗^*P* < 0.05.

After TNF-*α* stimulation, the IL-17A and IL-17A mRNA levels in fibroblasts significantly increased. After treatment with Wortmannin, the levels significantly decreased (Figures 5(a) and 5(b)).

## 4. Discussion

The etiology of CRSwNP is complex; patients usually require surgery, and recurrence is common. The pathogenesis remains unclear. Allergic CRSwNP usually does not respond well to treatment and often recurs after medication and surgery. In this study, six patients (13.3%) were positive in the Phadiatop test; no control was positive. Thus, systemic allergy plays a certain role in the pathogenesis of CRSwNP. Kennedy et al. found that patients with CRSwNP, particularly eosinophilic CRSwNP, were often positive in airborne allergen detection tests [6]. Erbek et al. studied 83 cases of CRSwNP and found that 55 (66.3%) were positive in skin prick tests [7]. Our findings are consistent with these reports.

Sinus CT is commonly used to image CRSwNP, revealing the extent of lesions that correlates with disease severity. However, Pallanch et al. reported a poor correlation between the CT Lund-MacKay score and subjective symptoms [8]. We found a correlation between the CT sinus score and only the serum level of total IgE, suggesting that the sinus score alone cannot serve as an objective indicator of CRSwNP disease severity. The CT score must be combined with other indicators.

The MPO activity reflects neutrophilic activation and inflammation. We found that MPO levels increased in nasal polyps, but the ECP and total IgE levels of the serum and tissues did not. Thus, neutrophilic inflammation may have been in play. Bachert et al. studied 93 Chinese patients with CRSwNP and found that only 7.5% exhibited eosinophilic inflammation [9]. Our results agree with this of previous work. We found an obvious positive correlation between systemic and local IgE production. Eckl-Dorna et al. found that the concentration of sIgE in peripheral blood mononuclear cells of patients with allergic inflammation was much lower than that of serum, suggesting that the sIgE of peripheral blood is produced principally in local tissues [10]. Our results are consistent with this suggestion; we found a close relationship between local and systemic IgE levels.

Hypoxia is closely related to CRSwNP development. Hypoxia can trigger nasal mucosa edema and persistent sinus inflammation [11]. One study found that the PI3K/Akt pathway became activated during hypoxia [12]. This pathway is closely associated with neutrophil activities; the downstream factors have major influences on neutrophil chemotaxis and survival. The PI3K/Akt signaling pathway plays an important regulatory role in allergic airway inflammation. However, any role for this pathway in CRSwNP has been less well studied. Park et al. found that the PI3K pathway regulated eosinophil recruitment to nasal polyp tissues [13]. We found that the nasal polyp levels of PI3K and p-Akt were significantly higher than the control values. Meanwhile, by the fibroblast experiments in vitro, we found that the activation of the PI3K/Akt pathway could increase the levels of IL-17A and IL-17A mRNA. The inhibition of the PI3K/Akt pathway could reduce the levels of IL-17A and IL-17A mRNA. We suggest that the PI3K/Akt pathway played a role in the pathogenesis of CRSwNP. Currently, the PI3K inhibitors Wortmannin and LY294002, and Akt inhibitors, are widely used to block the PI3K/Akt pathway. The PI3K/Akt pathway of noneosinophilic CRSwNP may serve as a novel therapeutic target for the disease.

The PI3K/Akt pathway lies upstream of HIF-1*α*, which is the key link between hypoxia and inflammation. CRSwNP is often associated with hypoxia, which elevates the HIF-1*α* level. Chien et al. used IHC and RT-PCR to show that the nasal polyp level of the HIF-1*α* protein increased, but the level of mRNA encoding HIF-1*α* mRNA did not [14]. Shi et al. found that the HIF-1*α* level was significantly increased in noneosinophilic CRSwNP and CRSsNP patients, associated with neutrophil infiltration [15]. Payne et al. used a microarray chip and RT-PCR to show that the HIF-1*α* level was significantly increased in noneosinophilic CRSwNP patients [16]. Luo et al. found that the HIF-1*α* protein and mRNA levels increased in both CRSwNP and CRSsNP patients [17]. We found that HIF-1*α* mRNA levels were significantly higher in nasal polyp groups than in controls, but HIF-1*α* protein levels were not. After TNF-*α* stimulation, both the HIF-1*α* protein and mRNA levels in fibroblasts were increased, but these significantly decreased after Wortmannin-mediated inhibition, indicating that HIF-1*α* plays a role in CRSwNP. The above results further confirm a role for the PI3K/Akt/HIF-1*α* pathway in the pathogenesis of CRSwNP. However, we found no significant increase in HIF-1*α* levels in nasal polyps, unlike a previous work [16]. We speculate that the PI3K/Akt pathway activation in CRSwNP patients only partially activates HIF-1*α* and other downstream factors such as NF-*κ*B.

Th17 cell numbers may correlate with CRSwNP pathogenesis. Shen et al. found a significant Th17/Treg imbalance (the Th17 cell proportion was increased) in CRSwNP patients [18]. IL-17A secreted by these cells plays roles in both innate and acquired immunity and upper respiratory tract diseases. IL-17A recruits and activates neutrophils to promote neutrophilic inflammation [19]. We found that the level of IL-17A mRNA was significantly higher in nasal polyp groups than in controls. It indicated that IL-17A plays a role in CRSwNP pathogenesis. After TNF-*α* stimulation, the fibroblast levels of IL-17A and IL-17A mRNA increased, but both significantly decreased after Wortmannin-induced inhibition. It implied that IL-17A levels were regulated by the PI3K/Akt/HIF-1*α* pathway. Combined with MPO upregulation, we speculate that IL-17A upregulation caused the neutrophilic inflammation that we observed. Ikejiri et al. found that T cell differentiation into Th17 cells was accelerated in a hypoxic environment and inhibited by the absence of HIF-1*α* [20], further indicating that HIF-1*α* may promote T cell differentiation into Th17 cells, a process supported by our results.

In conclusion, CRSwNP principally manifested as neutrophilic inflammation in this study. The PI3K/Akt/HIF-1*α* pathway plays a role in the pathogenesis of CRSwNP, as does IL-17A, the level of which is regulated by the PI3K/Akt/HIF-1*α* pathway. IL-17A upregulation may cause the neutrophilic inflammation of noneosinophilic CRSwNP.

The English in this document has been checked by at least two professional editors, both native speakers of English. For a certificate, please see http://www.textcheck.com/certificate/slEmMi.

## Figures and Tables

**Table 1:** Correlations among tissue indicators in CRSwNP patients.

Relevance (*r*)	ECP	Total IgE	MPO	p-Akt	PI3K	IL-17A mRNA	HIF-1*α* mRNA
ECP		-0.025	0.120	-0.020	-0.001	-0.064	0.003
Total IgE			0.089	-0.129	-0.131	-0.245	-0.113
MPO				-0.001	-	-0.116	-0.047
p-Akt					0.755	0.050	0.397
PI3K						-0.014	0.438
HIF-1*α*	-0.124	-0.081	-0.036	0.408	0.386	0.073	0.415
HIF-1*α* mRNA						0.059	

**Table 2:** The levels of HIF-1*α*, p-Akt, and PI3K protein in nasal polyp fibroblasts.

*β*-Actin	HIF-1*α*	p-Akt	PI3K
Fibroblasts	0.064 (0.044-0.086)	0.132 (0.125-0.192)	0.260 (0.227-0.357)
Fibroblasts + TNF-*α*	0.142 (0.086-0.251)	0.353 (0.298-0.605)	0.503 (0.378-0.805)
Fibroblasts + TNF-*α* + Wortmannin	0.078 (0.069-0.087)	0.254 (0.249-0.289)	0.292 (0.276-0.378)

## Data Availability

The datasets used and/or analyzed during the current study are available from the corresponding author on reasonable request.
